# Pediatric oncology services in Colombia

**DOI:** 10.25100/cm.v49i1.3377

**Published:** 2018-03-30

**Authors:** Amaranto Suarez Mattos, Jairo Aguilera, Edgar Augusto Salguero, Carolina Wiesner

**Affiliations:** 1 Instituto Nacional de Cancerología, Bogota, Colombia

**Keywords:** Medical care, comprehensive, children, cancer, Colombia, atención, integral, niños, cáncer, Colombia

## Abstract

In low-income countries, a child diagnosed with cancer has an 80% chance of dying, while in high-income countries more than 80% survive the disease. In Colombia, a middle-income country, the government issued new legislation that promotes the generation of comprehensive care units; nevertheless, seven years after its expedition, no institution has been recognized as such by the Ministry of Health. The objective of this study was to characterize the current offer of oncological services for cancer care in children and to identify the institutions that can be constituted in Units of Comprehensive Care of Childhood Cancer in Colombia.

**Methods:**

descriptive study of secondary source, the Special Register of Health Providers of the Ministry of Health and Social Protection was consulted, in order to identify the institutions that had enabled hospitalization services of medium or high complexity, chemotherapy, specialized consultation, emergencies, oncological surgery, and radiotherapy or nuclear medicine. The information is reported in absolute frequencies.

**Results:**

Seventy one institutions have hematology-oncology consultation, 39 institutions have chemotherapy and hospitalization services of medium or high complexity, and 18 have radiotherapy enabled. Only nine of the institutions include all the services that are necessary for comprehensive care.

**Conclusion:**

Colombia has a sufficient supply of services for the care of children with cancer. Only a minority are in institutions that have the capacity to guarantee the integrality of the attention.

## Resumen

**Antecedentes:**

En los países de bajos ingresos un niño diagnosticado con cáncer tiene un 80% de probabilidad de morir mientras que en los países de ingresos altos más del 80% sobrevive a la enfermedad. En Colombia, un país de ingresos medios, el gobierno expidió una nueva legislación que promueve la generación de unidades de atención integral; sin embargo, siete años después de su expedición, ninguna Institución ha sido reconocida como tal por el Ministerio de Salud.

**Objetivo:**

Caracterizar la actual oferta de servicios oncológicos para atención de cáncer en niños e identificar las instituciones que podrían ser constituirse en Unidades de Atención Integral de Cáncer Infantil en Colombia.

**Métodos:**

estudio descriptivo de fuente secundaria, se consultó el Registro Especial de Prestadores de Salud del Ministerio de Salud y Protección Social; identificando las instituciones que tenían habilitados servicio de hospitalización de mediana o alta complejidad, quimioterapia, consulta especializada, urgencias, cirugía oncológica, radioterapia o medicina nuclear. Se reporta la información en frecuencias absolutas.

**Resultados:**

Setenta y una instituciones cuentan con consulta de hemato-Oncología, 39 instituciones tienen servicios de quimioterapia y hospitalización de mediana o alta complejidad y 18 tienen habilitada radioterapia. Solo nueve de las instituciones incluyen la totalidad de los servicios necesarios para la atención integral.

**Conclusión:**

Colombia cuenta con una oferta suficiente de servicios para atención de niños con cáncer. Solo una minoría se encuentra en Instituciones que tienen la capacidad de garantizar la integralidad de la atención.

## Introduction

Cancer in children aged 0 to 14 is considered a rare disease, it represents between 0.5% to 2% of all cancer cases in the world. It is estimated that 200,000 new cases of cancer in children aged under 15 are diagnosed annually worldwide ^1^. 84% of children dying from cancer live in countries with low or intermediate income, where there is limited access to health care and cancer care ^2^. Clinical results differ substantially; while in low-income countries a child diagnosed with cancer has an 80% chance of dying, in high-income countries over 80% survive the disease ^3^ ^,^ ^4^.

In Colombia, cancer is the second cause of death after deaths for external causes in the group of 0 - 14 years of age ^5^. Each year, 1,322 new cases of cancer are diagnosed ^6^. In the five-year period 1990-95 the survival of children with Acute Lymphoblastic Leukemia in Colombia was 40.9%; and for the five-year period 2005-9, it was 53.8%. Although it shows an improvement, the results are much lower compared to other countries in America ^7^.

The differences in the results between the countries have a multifactorial origin that involves a series of factors, such as the social and economic determinants of each region ^6^ Taking into account that it is a pathology of low frequency and high complexity, health systems need to organize their offer of services to guarantee that access to the diagnosis and treatment of children with cancer is concentrated in institutions that have specialized human talent, biomedical technologies and the necessary infrastructure for the complexity of care. In this sense, twinning programs between hospitals located in countries with great experience and others located in low-income countries have allowed to improve survival ^8^ ^,^ ^9^.

In Colombia there have been multiple barriers to access timely treatments for children with cancer: the delay by insurers in the delivery of authorizations for care, the delay in the delivery of medicines, and the fragmentation of services and inter-institutional transfers to achieve comprehensive care ^10^ ^,^ ^11^. Due to these problems, the national government issued a new legislation as legal support to reduce cases of death due to cancer in children and persons aged under 18 ^12^ ^,^ ^13^. Colombian laws promote comprehensive treatment and they have delegated the Ministry of Social Protection to sector the services taking into account the demand needs and geographical location. They also created the National Advisory Council for Childhood Cancer to follow-up and monitor the implementation of these laws, as well as the national policies and plans that derive from it.

Within the framework of this regulation, the creation of Child Cancer Care Units (CCCU) was defined as units "located in hospitals or clinics of level III and IV of pediatric complexity, or with pediatric services of level III or IV" ^12^. The definition of the concept of UACAI transformed the model of habilitation of the pediatric oncological services towards a model in which the provider institutions must guarantee the services related to the care of children with cancer, in order to guarantee the integrality in the attention and the optimization of resources ^13^. However, seven years after its dissemination, the country does not have any UACAI recognized under that name by the Ministry of Health. In this sense, this article makes a descriptive analysis of the offer of institutions providing health services in Colombia that could be constituted as Units of Comprehensive Care CCCU with the purpose of promoting its implementation. 

## Materials and Methods

This is a descriptive operational study that uses the Special Registry of Health Providers (SRHP) of the Ministry of Health and Social Protection as a secondary source, consulted on August 29, 2016: This registry is permanently updated by the territorial health entities, which makes it changeable in time.

To identify the institutions that provide health services (hereafter referred as IPS) that can be constituted in CCCU, the technical annex "Manual of Habilitation" was taken from the Ministry of Health and Social Protection, which considers three central standards: organization or structure, management and health outcomes ^13^. The study defined in its scope of the first standard "Organization of the CCCU".

The Habilitation manual describes the services with which CCCU meets the requirement of "it may provide" and have available for its conformation: medium or high complexity hospitalization service and chemotherapy service. In the same way and as a criterion in the identification made in this study, the provider institutions that were enabled in the REPS services were taken into account such as: Specialized external consultation, emergencies, oncological surgery, radiotherapy or nuclear medicine.

The search was parameterized taking into account the following variables: group of services, service code, name of the provider, level of complexity, legal nature of the provider and province. The search was oriented to IPSs and not to independent providers; the search profile used was *guest*, the codes of the services consulted were: 391 oncology and pediatric hematology consultation, 374 pediatric surgery consultations, 227 pediatric oncological surgeries, 709 chemotherapy, 711 radiotherapy, 715 nuclear medicine, 102 pediatric general hospitalizations, 501 emergency services.

**Figure 1:**
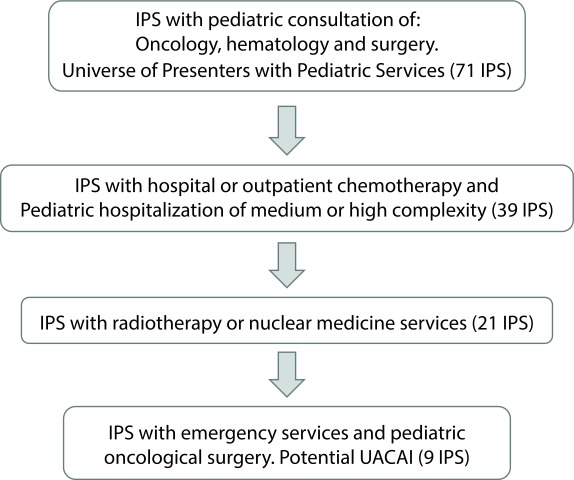
Algorithm describing the results of the number of health service providers (IPS) in each of the searches carried out.

The search strategy in the REPS focused on the following route:

1. Identification of the initial universe of providers that prescribe treatments in pediatric oncology: providers who had one of the following services enabled: pediatric hematology and oncology consultation and pediatric surgery consultation.

2. Identification of qualified chemotherapy services in any form of ambulatory or hospital care and pediatric hospitalization of medium or high complexity.

3. Identification of support services and therapeutic complementation of radiotherapy or nuclear medicine.

4. Identification of emergency services and pediatric oncological surgery. This last result was converted for the study into the final input that shows the potential number of institutions providing health services that can structure their services under a care strategy as CCCU (Fig. 1).

For the analysis there were simple frequencies obtained by province and by service. In a progressive manner, there were institutions excluded that did not have all the services that from a theoretical rather than a regulatory point of view should constitute an UACAI, such as pediatric hospitalization, outpatient oncology and pediatric hematology consultation and pediatric surgery consultation, chemotherapy in any form of ambulatory or hospital care and hospitalization, radiotherapy or nuclear medicine, emergencies and pediatric oncological surgery.

## Results

According to the SRHP as of the cutoff date of August 29, 2016, there were 71 Provider Institutions of Health Services identified, "Universe" of the country that has specialized consultation of oncology and hematology or consultation of oncological surgery for pediatric cancer care; they are distributed in 19 of the 32 provinces of the country. 69 IPS have a pediatric oncology and hematology consultation, and 11 also include oncological surgery (Table 1).

**Table 1:** Distribution by province of IPS with authorized services of pediatric oncological consultation, pediatric hospitalization of medium or high complexity and chemotherapy**.**

Province	Number of IPS*	IPS with Oncology and hematology	IPS with hospitalization and chemotherapy	IPS with oncologic surgery	IPS with both consultation services
Antioquia	6	6	4	0	0
Atlántico	13	12	5	2	1
Bogotá D.C**	13	12	9	4	3
Bolívar	3	3	3	1	1
Caldas	2	2	1	0	0
Cesar	2	2	NR	0	0
Córdoba	2	2	1	0	0
Huila	2	2	1	0	0
La Guajira	2	2	NR	0	0
Magdalena	1	1	NR	0	0
Meta	1	1	NR	0	0
Nariño	1	1	1	0	0
Norte de Santander	3	3	1	0	0
Quindío	1	1	1	0	0
Risaralda	4	4	3	0	0
Santander	4	4	3	2	2
Sucre	3	3	1	0	0
Tolima	1	1	1	0	0
Valle del Cauca	7	7	4	2	2
Total Colombia	71	69	39	11	9

Source: SRHP August 2016. Elaboration: self-made (study team)

IPS: Healthcare Provider Institution. * IPS with some service enabled under the SRHP service codes: 391, 374.

** Bogotá D.C. represents the Province of Cundinamarca.

The Province of Atlántico registers 13 institutions, a greater number than other Provinces that have capital cities with similar characteristics, such as Antioquia or Valle del Cauca, which register a lower number of IPS, six and seven respectively; that way Atlántico reports just the same number of IPS than the City of Bogotá D.C. (Bogotá represents the entire Province of Cundinamarca).

Among the 71 Healthcare Provider Institutions (IPS), 39 institutions distributed in 15 Provinces "have/may provide" chemotherapy and hospitalization services of medium or high complexity (Table 1). The Provinces that did not meet the search criteria for hospitalization services of medium and high complexity and chemotherapy were Cesar, La Guajira, Magdalena and Meta.

Of the 39 registered IPS with qualified hospitalization services of medium or high complexity and chemotherapy, the ones that had support services and therapeutic complementation of radiotherapy or nuclear medicine were verified, being identified a total of 21 IPS distributed in 11 Provinces (Table 2). As it can be seen, 18 IPS have enabled the radiotherapy service, 12 of them have enabled the nuclear medicine service and nine have the two services described above.

**Table 2:** Distribution by province of IPS with oncological consultation services, medium and high complexity pediatric hospitalization, chemotherapy, radiotherapy and/or nuclear medicine.

Province	Number of IPS	Radiotherapy	Nuclear Medicine	Both services
Antioquia	2	2	2	2
Atlántico	2	2	NR	NR
Bogotá D.C	6	3	6	3
Córdoba	1	1	NR	NR
Huila	1	1	NR	NR
Quindío	1	1	NR	NR
Risaralda	1	1	NR	NR
Santander	2	2	NR	NR
Sucre	1	1	1	1
Tolima	1	1	NR	NR
Valle del Cauca	3	3	3	3
**Total Colombia**	**21**	**18**	**12**	**9**

Source: SRHP August 2016. Elaboration: self-made (study team)

SRHP: Special Registry of Service Providers of the Ministry of Health and Social Protection

IPS: Healthcare Provider Institution

The number of institutions that met the requirements to establish themselves as CCCU were reviewed, that is to say, that they had the services to guarantee the integrality in the diagnosis and treatment of children with cancer. Table 3 shows that 9 of the IPS (located in the Provinces of Atlántico, Santander, Valle del Cauca and Bogotá City) met the criterion of concentrating the greatest number of services in the same physical space. In relation to this, however, only four of the nine IPS comply with pediatric oncological surgery offer, eight with radiotherapy and seven with nuclear medicine.

**Table 3:** Distribution by Province of IPS with oncological consultation services, hospitalization and chemotherapy, radiotherapy or nuclear medicine, pediatric surgery and emergency service.

Province	Number of IPS	Oncology and hematology	Oncologic surgery	Radiotherapy	Nuclear Medicine	Emergency services
Atlántico	2	2	0	2	2	2
Bogotá D.C	2	2	1	1	2	2
Santander	2	2	1	2	0	2
Valle del Cauca	3	3	2	3	3	3
**Total Colombia**	**9**	**9**	**4**	**8**	**7**	**9**

IPS: Healthcare Provider Institution. Source: SRHP August 2016. Elaboration: self-made (study team)

## Discussion

This study makes an analysis of the offer of pediatric cancer services in Colombia that fulfill the guarantees to establish a diagnosis and comprehensive treatment for patients with cancer. According to the study, it is found that of the 71 qualified institutions, that is, guaranteed to offer oncological services for children with cancer, only 21 of them have hospitalization, a chemotherapy room, a Hematology-oncology clinic and a pediatric oncological surgery clinic; and only 9 (12%) of the institutions are able to guarantee the integrality of that care in Colombia.

High-income countries have defined the criteria that cancer centers must fulfill in order to be able to offer care for inpatients and outpatients diagnosed with childhood cancer. Emphasis has been placed on the fact that the facilities must ensure timely accurate diagnosis, the administration of intensive chemotherapy, emergency management for serious complications 24 hours a day, intensive care services, and timely and complete blood support (blood bank) among others, and to have a network of hospitals that offer treatments as part of a shared attention ^14^ ^,^ ^15^ ^,^ ^16^. This shared network is important because the radiotherapy service is not always found within the hospitals, and this does not stop the care from being comprehensive, as long as the care is guaranteed if required, particularly for cases of central nervous system tumors. This is the case of the hospital network in Chile under the PINDA program ^16^.

Human talent is an essential requirement and institutions must have a multidisciplinary team led by pediatric hematologist/oncologists with the support of pediatricians, subspecialists in some areas of pediatrics, pediatric surgeons, intensive pediatricians, rehabilitators, nurses and other professionals ^13^ ^,^ ^14^. Since the number of cases of pediatric cancer is relatively low, the quality of treatment is guaranteed when the same institution receives a significant volume of children with cancer.

Likewise, there must be available educational programs for patients and family members, school programs, including contact with teachers who teach students at home or hospital, as well as support with reincorporation to school, and social support programs to help families with their concerns about economic difficulties and about the treatment and expenses that are going to be incurred ^17^.

Without compliance with these minimum conditions, it is very difficult for children, adolescents and young adults to benefit from the progress made in high income countries, due to the fact that an accurate diagnosis, adequate treatment, and medical and social support care depend on a multidisciplinary team and an infrastructure enabled in the institutions to treat cancer.

According to the present study, 19 out of the 32 Provinces of Colombia have a pediatric oncology service enabled, and these are concentrated in six provinces (Bogotá, Atlántico, Valle del Cauca, Antioquia, Santander and Risaralda) which is adequate taking into account that Cancer in children is a rare pathology. It is striking that the Province of Atlántico, with a population approximately four times smaller than the city of Bogotá, has the same number of institutions with oncology services enabled. As a possible explanation to this situation, it is found that most of the institutions that offer these services are private institutions that offer a broad service portfolio regardless of their ability to guarantee integral conditions in the care of children with cancer. In Colombia, the authorization of health services has been allowed, such as outpatient services, chemotherapy or hospitalization of children with cancer; without the need for them to be integrated within the same institution, which hardly guarantees a comprehensive and continuous care ^18^.

Faced with the objectives set in Colombia since 2010 ^11^, the goal of implementing comprehensive care for children with cancer has not been achieved. In the first place, it is found that the resolution defining the rating of UACAI was only published in July 2016 ^12^. Secondly, the authorization is voluntary, which means that the institutions do not have a motivation to do so, since a great effort to have all the required services is required. On the other hand, it is allowed that the UACAI be located in centers of "medium complexity," and that they may have services outside the same institution, which is a bit against the objective of having integral treatment centers; with the exception of the service of radiotherapy, which can certainly be shared by several institutions. This is how the regulation states, for example, that the hospitalization service may be available outside the (health) institution if it only has ambulatory surgery enabled ^12^.

A critical element that negatively affects the care of children and adolescents with cancer is that it allows potential CCCU not to have 24-hour emergency services in the pediatric hematology-oncology units as a requirement for habilitation, which is fundamental for the care of children. In this regard, Dang-tan *et al*. ^19^, reported on the delays in the diagnosis of pediatric solid tumors that, in general, the diagnosis was timelier when patients with suspicion of cancer were treated for the first time in an emergency service. On the other hand, and more importantly, is the need to have an immediate service for the complications caused by diseases or treatments that may endanger the lives of cancer patients.

Taking into account that the only source of information for performing this study was the REPS, there are some limitations because only information related to infrastructure could be included; the REPS does not allow to identify certain requirements demanded as "central of mixtures" or "program of pain and palliative care"; in the first case, the REPS does not identify these physical environments; in the second case, it does not identify programs. The fact of being the only source of information constitutes its main weakness. It is desirable to supplement the information with other primary sources. It is also possible, even though it is little feasible, for many providers to register authorized services that are not offering or that are inactive.

## Conclusion

It is found that Colombia has an adequate offer of oncological services for children with cancer; however, this offer only guarantees the integrality requirements in a small proportion. 
