# Supplementary material for: Pediatric oncology services in Colombia
Source: Colomb Med (Cali). 2018 Mar 30;49(1):97–101. doi: 10.25100/cm.v49i1.3377 (PMC6018828; doi:10.25100/cm.v49i1.3377)
Supplement: Related texts

# Research article: Servicios de oncología pediátrica en Colombia

## Introducción

El cáncer en los niños de 0 a 14 años de edad se considera una enfermedad rara que representa entre 0.5% a 2.0% de todos los canceres en el mundo; se estima que anualmente se diagnostican en el mundo 200,000 casos nuevos de cáncer en menores de 15 años ^1^. El 84% de esto niños viven en países con ingresos económicos bajos o intermedios donde existe un acceso limitado a los cuidados de la salud y a la atención oncológica ^2^. Los resultados clínicos difieren sustancialmente; mientras en los países de bajos ingresos un niño diagnosticado con cáncer tiene un 80% de probabilidad de morir, en países de ingresos altos más del 80% sobrevive a la enfermedad ^3^^,^^4^.

En Colombia, el cáncer constituye la segunda causa de muerte después de las muertes por causas externas en el grupo de 0-14 años ^5^. Cada año se diagnostican 1,322 casos nuevos de cáncer ^6^. En el quinquenio 1999-95 la supervivencia de niños con Leucemia Linfoide Aguda en Colombia fue de 40.9% y para el quinquenio 2005-9 fue de 53.8% que aunque muestra una mejoría, los resultado están muy por debajo comparado con otros países de América ^7^.

Las diferencias en los resultados entre los países tienen un origen multifactorial que involucra una serie de factores como son los determinantes sociales y económicas de cada región ^6^. Dado que se trata de una patología de baja frecuencia y alta complejidad los sistemas de salud requieren organizar la oferta de servicios para garantizar que el acceso al diagnóstico y tratamiento de los niños con cáncer se concentre en Instituciones que cuenten con el talento humano especializado, las tecnologías biomédicas y la infraestructura necesaria para la complejidad de la atención. En este sentido los programas de hermanamiento entre hospitales ubicados en países con gran experiencias y otros ubicados en países de menores ingresos ha permitido mejorar la supervivencia ^8^^,^^9^. 

En Colombia, se han registrado múltiples barreras para acceder a un tratamiento oportuno para los niños con cáncer: la demora en la entrega de autorizaciones para la atención por parte de los aseguradores, la demora en la entrega de medicamentos, la fragmentación de los servicios y los traslados interinstitucionales para lograr una atención integral ^10^^,^^11^. Frente a estos problemas el gobierno nacional expidió una nueva legislación como soporte jurídico para disminuir los casos de muerte a causa del cáncer en los niños y personas menores de 18 años ^12^. La Ley promueve el tratamiento integral y delegó al Ministerio de la Protección Social sectorizar la atención teniendo en cuenta las necesidades de la demanda y la ubicación geográfica. Igualmente creó el Consejo Nacional Asesor de Cáncer Infantil, para efectuar el seguimiento y monitoreo de la implementación de la presente ley, así como de las políticas y planes nacionales que de la misma se deriven. 

En el marco de esta reglamentación se definió la creación de Unidades de Atención de Cáncer Infantil (UACAI) como unidades “ubicadas en los hospitales o clínicas de nivel III y IV de complejidad pediátricos o con servicio de pediatría de nivel III o IV” (11). La definición del concepto de UACAI transformó el modelo de habilitación de los servicios oncológicos pediátricos hacia un modelo en el cual, las instituciones prestadoras deben garantizar los servicios relacionados con la atención de niños con cáncer para garantizar la integralidad en la atención y la optimización de recursos ^13^. Sin embargo, siete años después de su difusión aun el país no cuenta con UACAI reconocidas bajo tal denominación, por el Ministerio de Salud. En este sentido, este artículo hace un análisis descriptivo de la oferta de instituciones prestadoras de servicios de salud en Colombia que podrían constituirse como Unidades de Atención Integral de Cáncer Infantil UACAI con el propósito de promover su implementación. 

## Materiales y Métodos 

Se trata de un estudio descriptivo operativo que utiliza como fuente secundaria el Registro Especial de Prestadores de Salud (REPS) del Ministerio de Salud y Protección Social, consultado el 29 de agosto de 2016: Este registro es actualizado permanentemente por las entidades territoriales de salud, lo que lo hace cambiante en el tiempo. 

Para identificar las instituciones prestadoras de servicios de salud (IPS) que pueden constituirse en UACAI se tomó el anexo técnico “Manual de Habilitación” del Ministerio de Salud y Protección Social MSPS que considera tres estándares centrales: la organización o estructura, la gestión y los resultados en salud ^12^. El estudio, definió en su alcance el primer estándar “Organización de las UACAI”. 

El manual de Habilitación describe los servicios con que la UACAI con el requisito de “cuenta con” y “disponer de” para su conformación: servicio de hospitalización de mediana o alta complejidad y servicio de quimioterapia. De igual manera y como criterio en la identificación hecha en este estudio se tuvo en cuenta las instituciones prestadoras que tuvieran habilitados en el REPS servicios como: Consulta externa especializada, urgencias, cirugía oncológica, radioterapia o medicina nuclear, 

La búsqueda se parametrizó teniendo en cuenta las siguientes variables: grupo de servicios, código de servicio, nombre del prestador, nivel de complejidad, naturaleza jurídica del prestador y departamento. La búsqueda se orientó a instituciones prestadoras de salud (IPS) y no a prestadores independientes, el perfil de búsqueda utilizado fue el de invitado, los códigos de los servicios consultados fueron: 391 consulta de oncología y hematología pediátrica, 374 consulta de cirugía pediátrica, 227 cirugía oncológica pediátrica, 709 quimioterapia, 711 radioterapia, 715 medicina nuclear, 102 internación general pediátrica, 501 servicio de urgencias. 

La estrategia de búsqueda en el REPS siguió la siguiente ruta:

1. Identificación de universo inicial de prestadores que hacen prescripción de tratamiento en oncología pediátrica: prestadores que contaban con alguno de los siguientes servicios habilitados: consulta de oncología y hematología pediátrica y consulta de cirugía pediátrica. 

2. Identificación de servicios habilitados de quimioterapia en cualquiera de sus formas de atención ambulatoria u hospitalaria y hospitalización pediátrica de mediana o alta complejidad. 

3. Identificación de servicios de apoyo y complementación terapéutica de radioterapia o medicina nuclear. 

4. Identificación de los servicios de urgencias y cirugía oncológica pediátrica. Este último resultado se convirtió para el estudio en el insumo final que muestra el número potencial de instituciones prestadoras de servicios de salud que pueden estructurar sus servicios bajo la estrategia de atención como Unidad de Atención de Cáncer Infantil UACAI (Fig. 1). 

**Figure 1:** Algoritmo que describen los resultados del número de instituciones prestadoras de servicios de salud (IPS) en cada una de las búsquedas realizadas.

Para el análisis, se obtuvieron frecuencias simples por departamento y por servicio. De manera progresiva se fueron excluyendo las instituciones que no contaban con todos los servicios que desde el punto de vista teórico mas no reglamentario deben constituir una UACAI como son; hospitalización pediátrica, consulta externa de oncología y hematología pediátrica y consulta de cirugía pediátrica, quimioterapia en cualquiera de sus formas de atención ambulatoria u hospitalaria y hospitalización, radioterapia o medicina nuclear, urgencias y cirugía oncológica pediátrica. 

## Resultados

De acuerdo con el Registro Especial de Prestadores de Salud (REPS) a fecha al corte del 29 de Agosto de 2016 se identificaron 71 Instituciones Prestadoras de Servicios de Salud, “Universo” del país que cuentan con consulta especializada de oncología y hematología o consulta de cirugía oncológica para la atención de cáncer pediátrico distribuidas en 19 de los 32 Departamentos del país. Sesenta y nueve IPS tenían habilitada la consulta de oncología y hematología pediátrica y 11 incluyeron además cirugía oncológica (Tabla 1). 

**Tabla 1:** Distribución por departamento de IPS con servicios habilitados de consulta oncológica pediátrica, hospitalización pediátrica de mediana o alta complejidad y quimioterapia.

Departamento	Número de IPS*	IPS con Oncología y hematología	IPS con hospitalización y quimioterapia	IPS con Cirugía oncológica	IPS con ambas consultas
Antioquia	6	6	4	0	0
Atlántico	13	12	5	2	1
Bogotá D.C**	13	12	9	4	3
Bolívar	3	3	3	1	1
Caldas	2	2	1	0	0
Cesar	2	2	NR	0	0
Córdoba	2	2	1	0	0
Huila	2	2	1	0	0
La Guajira	2	2	NR	0	0
Magdalena	1	1	NR	0	0
Meta	1	1	NR	0	0
Nariño	1	1	1	0	0
Norte de Santander	3	3	1	0	0
Quindío	1	1	1	0	0
Risaralda	4	4	3	0	0
Santander	4	4	3	2	2
Sucre	3	3	1	0	0
Tolima	1	1	1	0	0
Valle del Cauca	7	7	4	2	2
**Total Colombia**	**71**	**69**	**39**	**11**	**9**

**Fuente:** REPS agosto de 2016, Elaboración propia equipo estudio

*IPS con algún servicio habilitado bajo los códigos de servicio REPS: 391, 374.

** Bogotá D.C. representa al Departamento de Cundinamarca.

El Departamento de Atlántico registró 13 instituciones, un número mayor respecto a otros Departamentos que cuentan con ciudades capitales con similares características como Antioquia o Valle del Cauca, los cuales registraron un menor número de IPS, 6 y 7 respectivamente; es así como Atlántico reportó incluso, el mismo número de IPS que la Ciudad de Bogotá D.C. (Bogotá representó a todo el Departamento de Cundinamarca).

Entre las 71 Instituciones Prestadoras de servicios de Salud (IPS) 39 instituciones distribuidas en 15 Departamentos “cuentan con” los servicios de quimioterapia y hospitalización de mediana o alta complejidad (Tabla 1). Los Departamentos que no cumplieron con el criterio de búsqueda para los servicios de hospitalización de mediana y alta complejidad y quimioterapia fueron Cesar, La Guajira, Magdalena y Meta. 

De las 39 IPS registradas con servicios habilitados de hospitalización de mediana o alta complejidad y quimioterapia se verificaron cuáles de ellas contaban con los servicios de apoyo y complementación terapéutica de radioterapia o de medicina nuclear, identificando un total de 21 IPS distribuidas en 11 Departamentos (Tabla 2). Como se puede observar 18 IPS tienen habilitado el servicio de radioterapia, 12 de estas tienen habilitado el servicio de medicina nuclear y 9 cuentan con los dos servicios antes descritos. 

**Tabla 2:** Distribución de IPS por departamento que cuentan con servicios de consulta oncológica, hospitalización pediátrica de mediana y alta complejidad, quimioterapia, radioterapia y/o medicina nuclear.

Departamento	Número de IPS	Radioterapia	Medicina Nuclear	Ambos servicios
Antioquia	2	2	2	2
Atlántico	2	2	NR	NR
Bogotá D.C	6	3	6	3
Córdoba	1	1	NR	NR
Huila	1	1	NR	NR
Quindío	1	1	NR	NR
Risaralda	1	1	NR	NR
Santander	2	2	NR	NR
Sucre	1	1	1	1
Tolima	1	1	NR	NR
Valle del Cauca	3	3	3	3
**Total Colombia**	**21**	**18**	**12**	**9**

**Fuente:** REPS agosto de 2016, Elaboración propia equipo estudio

REPS: Registro Especial de Prestadores de servicios del Ministerio de Salud y Protección Social

IPS: Institución Prestadora de Servicios de Salud

Se revisó el número de instituciones que cumplían con los requisitos para constituirse como UACAI es decir que contasen con los servicios para garantizan la integralidad en el diagnóstico y tratamiento del niño con cáncer. La Tabla 3 muestra que nueve de las IPS (ubicadas en los Departamentos de Atlántico, Santander, Valle del Cauca y La Ciudad de Bogotá) cumplirían el criterio de concentrar en un mismo espacio físico el mayor número de servicios. En relación con ello, sin embargo solo cuatro de las nueve IPS cumple con oferta de cirugía pediátrica oncológica, ocho con radioterapia y siete con medicina nuclear. 

**Tabla 3:** Distribución de IPS por departamento que cuentan con servicios de consulta oncológica, hospitalización y quimioterapia, radioterapia o medicina nuclear, cirugía pediátrica y servicio de urgencias.

Departamento	Número de IPS	Oncología y hematología	Cirugía Oncológica	Radioterapia	Medicina Nuclear	Urgencias
Atlántico	2	2	0	2	2	2
Bogotá D.C	2	2	1	1	2	2
Santander	2	2	1	2	0	2
Valle del Cauca	3	3	2	3	3	3
**Total Colombia**	**9**	**9**	**4**	**8**	**7**	**9**

**Fuente:** REPS agosto de 2016, Elaboración propia equipo estudio

## Discusión

Este estudio hace un análisis de la oferta de servicios oncológicos pediátricos en Colombia que cumplen con las garantías para poder establecer un diagnóstico y un tratamiento integral a los pacientes con cáncer. De acuerdo con el estudio, se encontró que de las 71 instituciones habilitadas, es decir, avaladas para ofrecer servicios oncológicos para niños con cáncer, solo 21 de ellas tiene hospitalización, sala de quimioterapia, consulta de Hemato-oncología y consulta de cirugía oncológica pediátrica y solo 9 (12%) de las instituciones están en capacidad de garantizar la integralidad de la atención en Colombia 

Los países de altos ingresos han definido los criterios que deben cumplir los centros oncológicos para estar en capacidad de ofrecer atención para pacientes hospitalizados y ambulatorios con diagnóstico de cáncer infantil. Se ha hecho énfasis en que las instalaciones deben garantizar la realización de un diagnóstico oportuno con precisión, la administración de quimioterapia intensiva, el manejo de urgencias para complicaciones graves durante las 24 horas del día, la atención en cuidados intensivos, el soporte hematológico oportuno y completo (banco de sangre) entre otros y, disponer de una red de hospitales que ofrezcan tratamientos como parte de una atención compartida ^14^^-^^16^. Esta red compartida es importante por cuanto el servicio de radioterapia no siempre se encuentra dentro de los hospitales y ello no impide que la atención sea integral siempre y cuando se garantice la atención en caso de requerirlo particularmente para los casos de tumores en el sistema nervioso central. Este es el caso de la red hospitalaria de Chile en el marco del programa PINDA ^16^.

El talento humano es un requerimiento imprescindible y las instituciones deben contar con un equipo multidisciplinario liderado por hemato-oncólogos pedíatras con el apoyo de médicos pediatras, subespecialistas en algunas áreas de pediatría, cirujanos pedíatras, pediatras intensivistas, rehabilitadores, enfermeras entre otros profesionales ^13^^,^^14^. Dado que el número de casos de cáncer pediátrico es relativamente bajo, la calidad del tratamiento se garantiza cuando una misma institución recibe un volumen importante de niños con cáncer. 

Igualmente se debe contar con programas educativos para el paciente y para los familiares, programas escolares, incluyendo contacto con los maestros, maestros que enseñan a estudiantes en el hogar o el hospital, así como apoyo con la reincorporación a la escuela y programas sociales de apoyo que ayuden a las familias con sus inquietudes sobre las dificultades económicas y sobre el tratamiento y los gastos que se incurran ^17^.

Sin el cumplimiento de estas condiciones mínimas es muy difícil que los niños, adolescentes y adultos jóvenes se beneficien de los avances logrados en los países con ingresos económicos altos, debido a que, el diagnóstico preciso, el tratamiento adecuado y los cuidados de apoyo médico y social dependen de un equipo multidisciplinario y una infraestructura habilitada en las instituciones para tratar el cáncer. 

De acuerdo con el presente estudio, de los 32 Departamentos de Colombia 19 tienen habilitado algún servicio oncológico pediátrico y estos están concentrados en seis departamentos del país (Bogotá, Atlántico, Valle del Cauca, Antioquia, Santander y Risaralda) lo cual es adecuado dado que el cáncer en niños es una patología poco frecuente. Llama la atención que el Departamento del Atlántico con una población aproximadamente cuatro veces menor a la ciudad de Bogotá tenga el mismo número de instituciones con servicios oncológicos habilitados. Como posible explicación a esta situación se encuentra que la mayoría de las instituciones que ofertan estos servicios son instituciones privadas que ofrecen un portafolio de servicio amplio independientemente de su capacidad de garantizar las condiciones de integralidad en la atención de los niños con cáncer. En Colombia, se ha permitido la habilitación de los servicios de salud, como servicios de consulta externa, o de quimioterapia o de hospitalización a niños con cáncer sin necesidad que estén integradas dentro de una misma institución lo cual difícilmente garantiza una atención integral y continua ^18^.

Frente a los objetivos planteados en Colombia desde el año 2010 ^11^ no ha logrado el objetivo de implementar la atención integral de niños con cáncer. En primer lugar, se encuentra que la resolución que define la habilitación de UACAI fue publicada solo hasta en Julio de 2016 ^12^. En segundo lugar, la habilitación es voluntaria lo cual hace que las instituciones no tengan una motivación para hacerlo pues exige un gran esfuerzo contar con todos los servicios requeridos. Por otra parte, se permite que la UACAI se ubique en centros de “mediana complejidad” y que puedan disponer de servicios por fuera de la misma institución lo que va un poco en contra del objetivo de tener centros integrales de tratamiento, con excepción del servicio de radioterapia que sin duda puede ser compartido por varias instituciones. Es así como la reglamentación plantea por ejemplo que el servicio de Hospitalización puede estar disponible por fuera de la Institución si tiene habilitado únicamente cirugía ambulatoria ^12^. 

Un elemento crítico que afecta negativamente la atención de los niños y adolescentes con cáncer es permitir que las potenciales UACAI no cuenten con servicios de urgencias las 24 horas en las unidades de hemato-oncología pediátrica como requisito para habilitación hecho que resulta fundamental para la atención de niños con cáncer. A este respecto y de manera adicional Tam Dang-tan *et al*. ^19^, en su estudio sobre las demoras en el diagnóstico de los tumores sólidos pediátricos informa que en general el diagnóstico fue más oportuno cuando los pacientes con sospecha de cáncer son atendidos por primera vez en un servicio de urgencias. Por otro lado, y más importante aún, es la necesidad de contar con un servicio de atención inmediata de las complicaciones producidas por la enfermedad y por el tratamiento que pone en peligro la vida del paciente.

Teniendo en cuenta que la única fuente de información para la realización de este estudio fue el REPS se encuentran algunas limitaciones pues solo se pudo incluir información relacionada con la infraestructura; el REPS no permite identificar ciertos requisitos exigidos como “central de mezclas” o “programa de dolor y cuidado paliativo”; en el primer caso el REPS no permite identificar estos ambientes físicos y en el segundo caso no identifica programas. El hecho de ser la única fuente de información se constituye en su principal debilidad. Es deseable complementar la información con otras fuentes primarias. Es posible también, aunque sea poco factible, que muchos prestadores registren servicios habilitados que no estén prestando o estén inactivos.

## Conclusión

Se encuentra que Colombia cuenta con una adecuada oferta de servicios oncológicos para niños con cáncer, sin embargo esta oferta solo en una menor proporción garantiza los requerimientos de integralidad.
